# Noninvasive Measurement of Carbon Dioxide during One-Lung Ventilation with Low Tidal Volume for Two Hours: End-Tidal versus Transcutaneous Techniques

**DOI:** 10.1371/journal.pone.0138912

**Published:** 2015-10-14

**Authors:** Hong Zhang, Dong-Xin Wang

**Affiliations:** Department of Anesthesiology and Critical Care Medicine, Peking University First Hospital, Beijing, China; Massachusetts General Hospital, UNITED STATES

## Abstract

**Background:**

There may be significant difference between measurement of end-tidal carbon dioxide partial pressure (PetCO_2_) and arterial carbon dioxide partial pressure (PaCO_2_) during one-lung ventilation with low tidal volume for thoracic surgeries. Transcutaneous carbon dioxide partial pressure (PtcCO_2_) monitoring can be used continuously to evaluate PaCO_2_ in a noninvasive fashion. In this study, we compared the accuracy between PetCO_2_ and PtcCO_2_ in predicting PaCO_2_ during prolonged one-lung ventilation with low tidal volume for thoracic surgeries.

**Methods:**

Eighteen adult patients who underwent thoracic surgeries with one-lung ventilation longer than two hours were included in this study. Their PetCO_2_, PtcCO_2_, and PaCO_2_ values were collected at five time points before and during one-lung ventilation. Agreement among measures was evaluated by Bland-Altman analysis.

**Results:**

Ninety sample sets were obtained. The bias and precision when PtcCO_2_ and PaCO_2_ were compared were 4.1 ± 6.5 mmHg during two-lung ventilation and 2.9 ± 6.1 mmHg during one-lung ventilation. Those when PetCO_2_ and PaCO_2_ were compared were -11.8 ± 6.4 mmHg during two-lung ventilation and -11.8 ± 4.9 mmHg during one-lung ventilation. The differences between PtcCO_2_ and PaCO_2_ were significantly lower than those between PetCO_2_ and PaCO_2_ at all five time-points (p < 0.05).

**Conclusions:**

PtcCO_2_ monitoring was more accurate for predicting PaCO_2_ levels during prolonged one-lung ventilation with low tidal volume for patients undergoing thoracic surgeries.

## Introduction

Arterial carbon dioxide partial pressure (PaCO_2_) is the gold standard in monitoring ventilation during general anesthesia. End-tidal carbon dioxide partial pressure (PetCO_2_) reflects PaCO_2_ and becomes a standard monitoring during surgery. However, various pathologic processes of the cardio-respiratory system such as ventilation-perfusion mismatch or shunt as well as changes in patient positioning have been shown to influence the correlation between PaCO_2_ and PetCO_2_ [1]. One-lung ventilation (OLV) and lateral decubitus position during thoracic surgery impair ventilation-perfusion matching and, as a result, the difference between PaCO_2_ and PetCO_2_.

Transcutaneous carbon oxide partial pressure (PtcCO_2_) monitoring provides a noninvasive and continuous estimation of PaCO_2_ by sampling from arterialized capillary blood and is not influenced by ventilation-perfusion mismatch [2]. Previous studies [3–4] found that during short time OLV (≤ 1 hour) for thoracic surgery, the value of PtcCO_2_ is closer to PaCO_2_ than PetCO_2_.

With the wide-spread use of mini-invasive thoracic surgery and the introduction of lung-protective ventilation strategy during thoracic anesthesia, prolonged hypercapnia originated from low tidal volume OLV in these patients are not uncommon [5–6]. We designed this study to evaluate the accuracy of PtcCO_2_ in predicting PaCO_2_ values during prolonged OLV and permissive hypercapnia during mini-invasive thoracic surgery.

## Materials and Methods

The study protocol was approved by the Clinical Research Ethics Committee of Peking University First Hospital (2012[504]). Written informed consent was obtained from each patient.

Eighteen adult patients of ASA physical status I or II who were scheduled to undergo mini-invasive thoracic surgery with an expected OLV duration of two hours or more were recruited for this study. Patients with diagnosed cardiovascular disease were excluded.

No premedication was administrated. Before the induction of general anesthesia, an epidural puncture was performed between the fifth and eighth thoracic interspace and an epidural catheter was inserted. A test dose of 3 ml 1% lidocaine was administered and no other epidural medication was used during anesthesia.

Intraoperative monitoring included a non-invasive blood pressure, pulse oxygen saturation, an electrocardiogram, nasopharyngeal temperature, urine output, peak airway pressure, and direct arterial blood pressure measurement through a radial artery catheter. General anesthesia was induced with propofol (1–2 mg/kg) and remifentanil (effect site target control infusion at a target of 4–6 ng/ml). And rocuronium (0.6mg/kg) was administered to facilitate endotracheal intubation with a double-lumen tube by direct laryngoscopy. Patients were mechanically ventilated in a volume-controlled manner both in the supine and the lateral decubitus position. During two-lung ventilation (TLV), the fresh gas flow, tidal volume, respiratory rate and inspiratory/expiratory ratio were set at 1 L/min oxygen and 1 L/min air, 6–8 ml/kg, 10–12 breath/min, and 1:2, respectively. Anesthesia was maintained with sevoflurane inhalation (end-tidal anesthetic concentration of 0.8 MAC or above) and remifentanil infusion until the end of the surgery. Sufentanil was administered as a bolus when deemed necessary during surgery and before the end of surgery.

For all patients, the position of the double-lumen endobronchial tube was confirmed under direct vision with a fiberoptic bronchoscope (FOB). The patients were then turned to the lateral decubitus position. The tube position was then checked again with the FOB just before OLV, and the effectiveness of lung collapse during OLV was confirmed by direct observation in the operative field. During OLV, the fresh gas flow, tidal volume, respiratory rate and inspiratory/expiratory ratio were set at 1 L/min oxygen, 4–6 ml/kg, 10–16 breath/min, and 1:1.5, respectively, to maintain a SpO_2_ of 90% or higher and a peak airway pressure lower than 25 cmH_2_O. Intravenous ephedrine or phenylephrine or nicardipine was administrated to maintain blood pressure fluctuation within 30% from baseline. Additional doses of rocuronium were administered to maintain muscle relaxation.

PtcCO_2_ was measured with a TCM3 transcutaneous CO_2_/oxygen device (Radiometer, Copenhagen, Denmark). The monitoring technique was standardized by applying the probe to the upper part of the patient’s dependent arm in the lateral decubitus position. Before each study, the device was calibrated by using a two-point self-calibration (5% and 10% CO_2_) and the working temperature of the electrode was maintained at 42°C to “arterialize” the skin capillary blood flow according to the manufacturer’s recommendations. The monitor used an internal adjustment to compensate for the effects of the heated probe on CO_2_ tension. It took appropriately 20 minutes for initial stabilization after probe attachment. The end-tidal concentrations of the anesthetics and CO_2_ were measured with a AS/5 monitor (Datex-Ohmeda, Helsinki, Finland) which was calibrated in 5% CO_2_ and 20.9% oxygen gas before the study. Continuous sampling was obtained from a connector attached to the proximal end of heat moisture exchanger in the respiratory circuit. Arterial blood samples were obtained during TLV, just before the initiation of OLV, and every 30 minutes during OLV until 120 minutes. Arterial blood gas analysis was performed using a GEM premier 3000 analyzer (Instrumentation Laboratory, USA). Data of PtcCO_2_, PetCO_2_, and PaCO_2_ monitoring results were collected simultaneously. The heart rate, mean arterial pressure, pulse oxygen saturation, and nasopharyngeal temperature were also recorded at same time-points.

Quantitative data were presented as means ± standard deviation (SD). Bland-Altman method was used to analyze the agreement between PaCO_2_ and PetCO_2_ or between PaCO_2_ and PtcCO_2_. The bias (the mean difference between the values) and the precision (the SD of the bias) were calculated. Student’s unpaired t-tests were also used to compare the differences between PaCO_2_ and PetCO_2_ or between PaCO_2_ and PtcCO_2_. A p value of less than 0.05 was regarded as statistically significant. Statistical analysis was conducted using SPSS version 14.0 (Chicago, IL, USA).

## Results

All eighteen patients completed the study protocol. The demographic data were shown in Table 1. Surgical procedures included lobectomy or pneumonectomy for lung cancer, and thymectomy for thymoma. A total of 90 data sets consisting of the simultaneous measurements of PtcCO_2_, PetCO_2_ and PaCO_2_ at five time points were obtained (Table 2). The heart rate and mean arterial pressure did not significantly change from the preoperative values during the study period. The body temperature remained constant between 35.5 and 36.5°C.

**Table 1:** Demographic data (n = 18).

Variable	Data
Age (yr)	59 ± 13
Sex (M/F)	13/5
Weight (kg)	61 ± 10
Height (cm)	165 ± 5
ASA classification I/II (n)	2/16
Smoking (n)	10

Values are expressed as mean ± standard deviation or number of patients.

**Table 2:** Carbon dioxide level in five time points.

Variable	TLV	OLV30	OLV60	OLV90	OLV120
PaCO_2_ (mmHg)	46.5 ± 6.9	52.2 ± 9.1	52.2 ± 7.0	52.4 ± 6.9	52.2 ± 6.6
PetCO_2_ (mmHg)	34.7 ± 4.7	39.7 ± 5.6	40.2 ± 4.8	41.2 ± 5.6	40.7 ± 5.5
PtcCO_2_ (mmHg)	50.6 ± 8.0	56.1 ± 10.5	55.2 ± 8.4	55.0 ± 10.7	54.4 ± 10.0

Values are expressed as mean ± standard deviation. OLV30, OLV60, OLV90 and OLV120 refer to 30, 60, 90 and 120 minutes of OLV, respectively. TLV, two-lung ventilation; OLV, one-lung ventilation.

**Fig 1:**
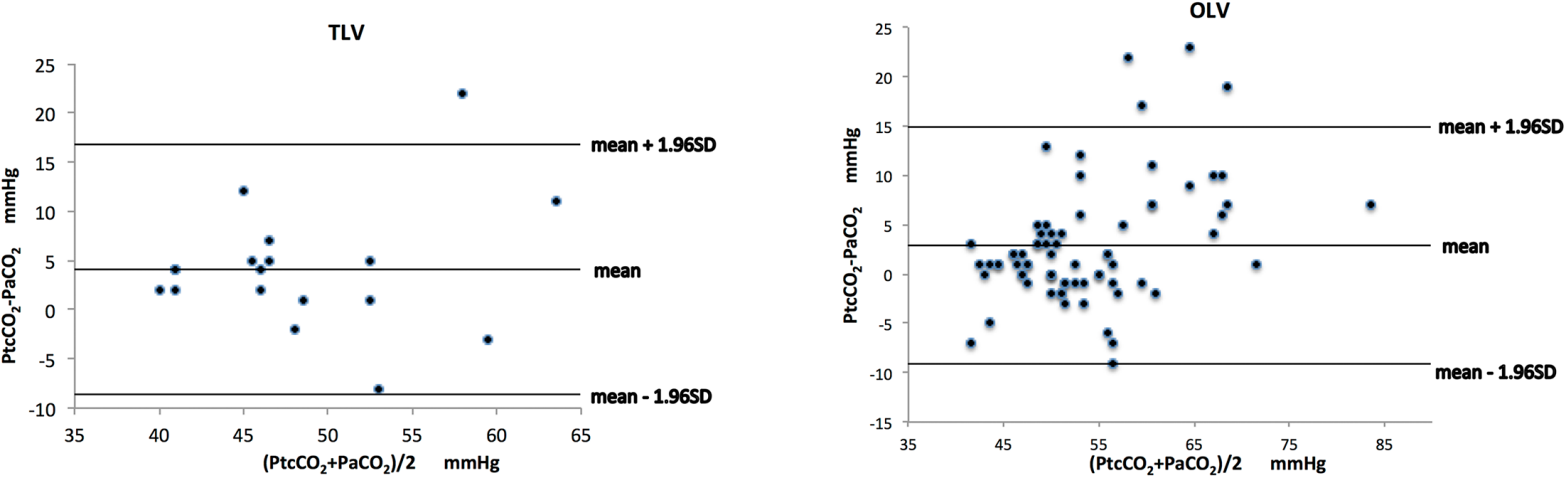
Agreement between transcutaneous CO_2_ (PtcCO_2_) and arterial CO_2_ (PaCO_2_). Bland-Altman analysis of PtcCO_2_ versus PaCO_2_ during two-lung ventilation (TLV) and one-lung ventilation (OLV). Bias was labeled. The 95% limits of agreement of the average PtcCO_2_ –PaCO_2_ difference during TLV and OLV were 4.1 ± 6.5 mmHg and 2.9 ± 6.1 mmHg (mean ± 1.96 standard deviation), respectively.

**Fig 2:**
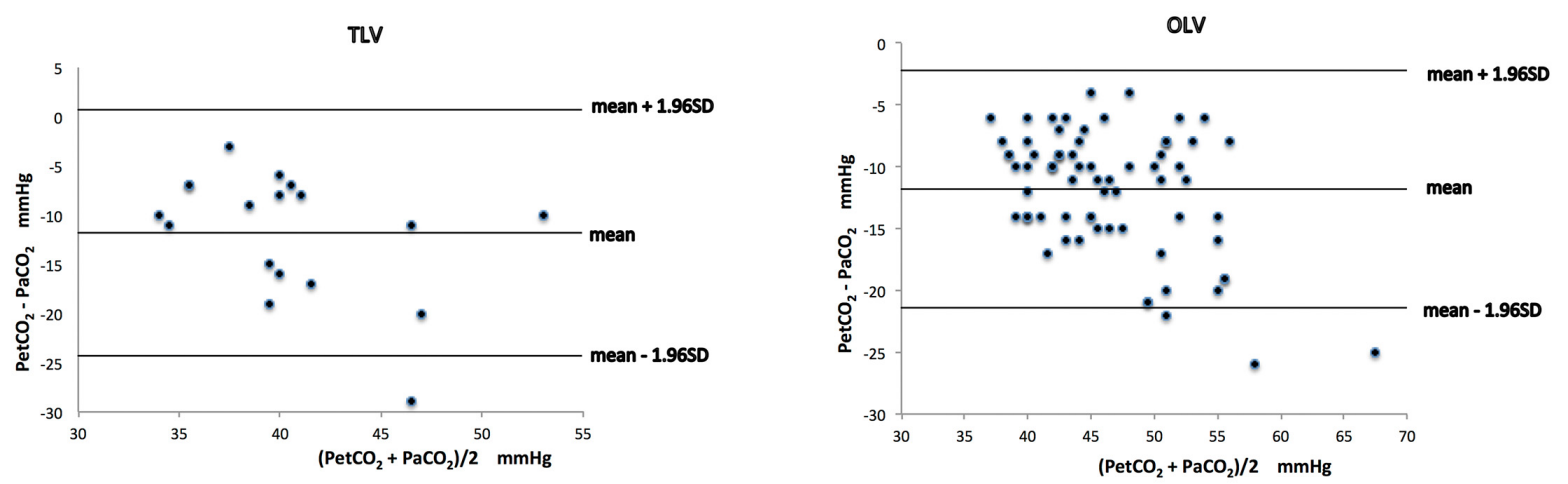
Agreement between end-tidal CO_2_ (PetCO_2_) and arterial CO_2_ (PaCO_2_). Bland-Altman analysis of PetCO_2_ versus PaCO_2_ during two-lung ventilation (TLV) and one-lung ventilation (OLV). Bias was labeled. The 95% limits of agreement of the average PetCO_2_ –PaCO_2_ difference during TLV and OLV were -11.8 ± 6.4 mmHg and -11.8 ± 4.9 mmHg (mean ± 1.96 standard deviation), respectively.

When PtcCO_2_ and PaCO_2_ were compared, the bias and precision were 4.1 ± 6.5 mmHg during TLV and 2.9 ± 6.1 mmHg during OLV, respectively (Fig 1). When PetCO_2_ and PaCO_2_ were compared, the bias and precision were -11.8 ± 6.4 mmHg during TLV and -11.8 ± 4.9 mmHg during OLV, respectively (Fig 2). The values of bias and precision were stable and the difference between PtcCO_2_ and PaCO_2_ was significantly lower than that between PetCO_2_ and PaCO_2_ throughout the 2-hour period of OLV (Table 3).

**Table 3:** Bland-Altman analysis at different time points.

Variable	TLV	OLV30	OLV60	OLV90	OLV120
PtcCO_2_-PaCO_2_ difference (mmHg)	4.1 ± 6.5*	3.8 ± 5.8*	2.9 ± 5.1*	2.6 ± 6.7*	2.2 ± 7.2*
PetCO_2_-PaCO_2_ difference (mmHg)	-11.8 ± 6.4	-12.5 ± 5.0	-12.0 ± 5.2	-11.3 ± 4.5	-11.5 ± 5.1

Values are expressed as bias ± precision. OLV30, OLV60, OLV90 and OLV120 refer to 30, 60, 90 and 120 minutes of OLV, respectively. TLV, two-lung ventilation; OLV, one-lung ventilation.

* Significantly different from PetCO_2_, PaCO_2_ differences (p < 0.05).

## Discussion

This study demonstrates that PtcCO_2_ monitoring is a more accurate estimation of PaCO_2_ than PetCO_2_ during OLV of two hours or more with low tidal volume for thoracic surgery. Our results are consistent with previous ones on the accuracy of PtcCO_2_ monitoring during thoracic surgery of shorter duration [3–4]. Moreover, we find that the PtcCO_2_ is accurate of in estimating the PaCO_2_ in the clinical condition of permissive hypercapnia which is not observed in previous studies [3–4].

PtcCO_2_ monitoring used in the present study is a non-invasive method for continuous measurement of transcutaneous CO_2_ partial pressure. It uses skin electrodes to quantify the amount of CO_2_ that diffuses to the electrode on the surface of the skin. Local heating is required in order to increase the local blood circulation in the capillary bed below the sensor. The accuracy of PtcCO_2_ monitoring is influenced by several factors including methodological limitations (e.g., stabilization time, reaction time, periodic repositioning of the sensor, the need for membrane restoration, and baseline calibration), technical mistakes (e.g., improper application of the sensor, trapped air bubbles in the electrolyte solution, damage to the sensor membrane, improper calibration) and hypoperfusion (e.g., vasoconstriction, hypothermia, shock, low cardiac output or local edema) [7].

Since its introduction into clinical practice, PtcCO_2_ monitoring has received great attention in neonates [8–9]. Later on, with the improvement of technology and increasing knowledge of this monitoring modality, its use has been increasing in pediatric patient [10–11], in adult patients undergoing thoracic [3–4] and laparoscopic surgery [12–15] and in patients after surgery [16–17]. The accuracy of PtcCO_2_ monitoring has been confirmed by these studies. However, some others reported that PtcCO_2_ monitoring cannot precisely predict PaCO_2_ in preterm infants during in their first 24 hours [18] and in some patients receiving artificial ventilation during general anesthesia [19], possibly because of the aforementioned reasons. Considering the relative high price and the failure possibility, appropriate indication should be considered in chosing this monitoring method. One suitable clinical condition for PtcCO_2_ monitoring is OLV during thoracic surgery.

OLV is essential for the success of mini-invasive thoracic surgery. Advances in this technique have enabled more complex intrathoracic procedures being performed. Protective ventilation strategy [5–6] is now widely used during OLV and consists of small tidal volumes, low inspired oxygen fraction, low airway pressures, permissive hypercapnia, and positive end expiratory pressure. Our results demonstrated the applicability of PtcCO_2_ during a 2-hour period of OLV with hypercapnia for thoracic surgery. The study of Oshibuchi et al. [3] found that PtcCO_2_ monitoring provides a more accurate estimation of PaCO_2_ than PetCO_2_ during an 1-hour period of OLV with normocapnia (PaCO_2_ in the range of 30–50 mmHg) for thoracic surgery. In another study, Kelly et al. [20] reported that the agreement between PtcCO_2_ and PaCO_2_ deteriorated at high PaCO_2_ levels (>60 mmHg). Our results extrapolate these ranges, i.e., a 2-hour period of OLV with hypercapnia (PaCO_2_ in the range of 35–70 mmHg) during thoracic surgery. Long time PtcCO_2_ monitoring (longer than 2 hours) was only observed in nonsurgical patients in previous studies [21–22].

A difference of 5 mmHg or less between PaCO_2_ and other carbon dioxide measurement was a clinically acceptable discrepancy [4,13]. Our results found that the bias between PtcCO_2_ and PaCO_2_ was less than 5 mmHg during either TLV or OLV, whereas the bias between PetCO_2_ and PaCO_2_ was lower than -11 mmHg during either TLV or OLV. In the scatter diagram of PetCO_2_ and PaCO_2_, 3 of 90 points were outside of the limits of agreement during OLV and all 3 points were beyond 22 mmHg. Whereas in the scatter diagram of PtcCO_2_ and PaCO_2_, 4 of 90 points were outside of the limits but only 1 point was beyond 22 mmHg. These also indicated the superiority of PtcCO_2_ in predicting PaCO_2_.

PetCO_2_ monitoring is still the most convenient method in CO_2_ measurement and has a unique role in judging the position of artificial airway and ventilation status. The difference between PaCO_2_ and PetCO_2_ increases with age, pulmonary disorders, pulmonary embolism, reduced cardiac output, hypervolemia, anesthesia, and other conditions that increase the ventilation-perfusion mismatch. In the present study, the mean difference between PetCO_2_ and PaCO_2_ during either TLV or OLV was higher than the previously reported ones in similar patients [3–4]. This is perhaps because the tidal volume settings during TLV (6–8 ml/kg) and OLV (4–6ml/kg) were lower in our study than in previous one (10 ml/kg throughout the operation) [3]. According to respiratory physiology, the ratio of dead space to tidal volume determines the gradient between PaCO_2_ and PetCO_2_ assuming that the PaCO_2_ remains constant. The higher difference between PetCO_2_ and PaCO_2_ in our study supports the use of PtcCO_2_ monitoring during prolonged OLV with lung-protective strategy of low tidal volume ventilation.

## Conclusions

In conclusion, our study demonstrated that PtcCO_2_ is more accurate than PetCO_2_ in estimating PaCO_2_ during prolonged OLV with low tidal volume ventilation for thoracic surgery.

## Supporting Information

S1 Dataset Data for analysis. (XLSX)

## References

[1] Whitesell R, Asiddao C, Gollman D, Jablonski J. Relationship between arterial and peak expired carbon dioxide pressure during anesthesia and factors influencing the difference. Anesth Analg 1981;60:508–12. 6787952

[2] Huttmann SE, Windisch W, Storre JH. Techniques for the measurement and monitoring of carbon dioxide in the blood. Ann Am Thorac Soc 2014; 11:645–52. 10.1513/AnnalsATS.201311-387FR 24701974

[3] Oshibuchi M, Cho S, Hara T, Tomiyasu S, Makita T, Sumikawa K. A comparative evaluation of transcutaneous and end-tidal measurements of CO_2_ in thoracic anesthesia. Anesth Analg 2003; 97:776–9. 12933401 10.1213/01.ANE.0000074793.12070.1E

[4] Tobias JD. Noninvasive carbon dioxide monitoring during one-lung ventilation: End-Tidal versus transcutaneous techniques. J Cardiothorac Vasc Anesth 2003; 17:306–8. 12827576 10.1016/s1053-0770(03)00054-5

[5] Kilpatrick B, Slinger P. Lung protective strategies in anaesthesia. Br J Anaesth 2010; 105 Suppl 1:i108–16. 10.1093/bja/aeq299 21148650 PMC9149602

[6] Brassard CL, Lohser J, Donati F, Bussieres JS. Step-by-step clinical management of one-lung ventilation: continuing professional development. Can J Anesth 2014; 61:1103–21. 10.1007/s12630-014-0246-2 25389025

[7] Eberhard P. The design, use, and results of transcutaneous carbon dioxide analysis: Current and future directions. Anesth Analg 2007; 105(6 Suppl):S48–52. 18048898 10.1213/01.ane.0000278642.16117.f8

[8] Binder N, Atherton H, Thorkelsson T, Hoath SB. Measurement of transcutaneous carbon dioxide in low birthweight infants during the first two weeks of life. Am J Perinatol 1994; 11:237–41. 8048993 10.1055/s-2008-1040754

[9] Bernet V, Döll C, Cannizzaro V, Ersch J, Frey B, Weiss M. Longtime performance and reliability of two different ptcCO_2_ and spOO_2_ sensors in neonates. Paediatr Anaesth 2008; 18:872–7. 10.1111/j.1460-9592.2008.02661.x 18768047

[10] Dullenkopf A, Bernardo SD, Berger F, Fasnacht M, Gerber AC, Weiss M. Evaluation of a new combined spOO_2_/ptcCO_2_ sensor in anaesthetized paediatic patients. Paediatr Anaesth 2003; 13:777–84. 14617118 10.1046/j.1460-9592.2003.01146.x

[11] Tobias JD. Transcutaneous carbon dioxide monitoring in infants and children. Paediatr Anaesth 2009; 19:434–44. 10.1111/j.1460-9592.2009.02930.x 19236597

[12] Bhavani-Shankar K, Steinbrook RA, Mushlin PS, Freiberger D. Transcutaneous PCO_2_ monitoring during laparoscopic cholecystectomy in pregnancy. Canadian Journal of Anaesthesia 1998; 45:164–9 9512853 10.1007/BF03013257

[13] Xue Q, Wu X, Jin J, Yu B, Zheng M. Transcutaneous carbon dioxide monitoring accurately predicts arterial carbon dioxide partial pressure in patients undergoing prolonged laparoscopic surgery. Anesth Analg 2010; 111:417–20 10.1213/ANE.0b013e3181e30b54 20584872

[14] Liu S, Sun J, Chen X, Yu Y, Liu X, Liu C. The application of transcutaneous CO_2_ pressure monitoring in the anesthesia of obese patients undergoing laparoscopic bariatric surgery. PLoS One 2014; 9(4):e91563 10.1371/journal.pone.0091563 24699267 PMC3974655

[15] De Oliveira GS, Ahmad S, Fitzgerald PC, McCarthy RJ. Detection of hypoventilation during deep sedation in patients undergoing ambulatory gynaecological hysteroscopy: A comparison between transcutaneous and nasal end-tidal carbon dioxide measurements. Br J Anaesth 2010; 104:774–8. 10.1093/bja/aeq092 20418266

[16] Hirabayashi M, Fujiwara C, Ohtani N, Kagawa S, Kamide M. Transcutaneous PCO_2_ monitors are more accurate than end-tidal PCO_2_ monitors. J Anesth 2009; 23:198–202. 10.1007/s00540-008-0734-z 19444557

[17] Roediger R, Beck-Schimmer B, Theusinger OM, Rusch D, Seifert B, Spahn DR, et al The revised digital transcutaneous PCO_2_/spOO_2_ ear sensor is a reliable noninvasive monitoring tool in patients after cardiac surgery. J Cardiothorac Vasc Anesth 2011; 25:243–9. 10.1053/j.jvca.2010.06.021 20851636

[18] Aliwalas LL, Noble L, Nesbitt K, Fallah S, Shah V, Shah PS. Agreement of carbon dioxide levels measured by arterial, transcutaneous and end tidal methods in preterm infants < or = 28 weeks gestation. J Perinatol 2005; 25:26–9. 15496874 10.1038/sj.jp.7211202

[19] Nishiyama T, Nakamura S, Yamashita K. Effects of the electrode temperature of a new monitor, TCM4, on the measurement of transcutaneous oxygen and carbon dioxide tension. J Anesth 2006; 20:331–4. 17072703 10.1007/s00540-006-0422-9

[20] Kelly AM, Klim S. Agreement between arterial and transcutaneous PCO_2_ in patients undergoing non-invasive ventilation. Respir Med 2011; 105:226–9. 10.1016/j.rmed.2010.11.010 21131188

[21] Storre JH, Steurer B, Kabitz HJ, Dreher M, Windisch W. Transcutaneous PCO_2_ monitoring during initiation of noninvasive ventilation. Chest 2007; 132:1810–6. 18079217 10.1378/chest.07-1173

[22] Janssens JP, Laszlo A, Uldry C, Titelion V, Picaud C, Michel JP. Non-invasive (transcutaneous) monitoring of PCO_2_ (tcpCO_2_) in older adults. Gerontology 2005; 51:174–8. 15832044 10.1159/000083990

